# An iTRAQ-Based Comparative Proteomics Analysis of the Biofilm and Planktonic States of *Aeromonas veronii* TH0426

**DOI:** 10.3390/ijms21041450

**Published:** 2020-02-20

**Authors:** Ying Li, Bintong Yang, Jiaxin Tian, Wuwen Sun, Guiqin Wang, Aidong Qian, Chunfeng Wang, Xiaofeng Shan, Yuanhuan Kang

**Affiliations:** 1 Key Laboratory of Animal Production and Product Quality Safety of Ministry of Education, College of Animal Science and Technology, Jilin Provincial Engineering Research Center of Animal Probiotics, Jilin Agricultural University, Changchun 130118, China; ly18943115236@163.com (Y.L.); gracebintong@163.com (B.Y.); 17767733220@163.com (J.T.); asdfghj123op@126.com (W.S.); wgqjlau2019@sina.com (G.W.); qianaidong0115@163.com (A.Q.); wangchunfeng@jlau.edu.cn (C.W.); 2 College of Life Science, Changchun Sci-Tech University, Changchun 130118, China

**Keywords:** *Aeromonas veronii*, biofilm, quantitative proteomics, iTRAQ

## Abstract

*Aeromonas veronii* is a virulent fish pathogen that causes extensive economic losses in the aquaculture industry worldwide. In this study, a virulent strain of *A. veronii* TH0426 was used to establish an in vitro biofilm model. The results show that the biofilm-forming abilities of *A. veronii* TH0426 were similar in different media, peaking under conditions of 20 °C and pH 6. Further, isobaric tags for relative and absolute quantitation (iTRAQ)-based quantitative proteomics methods were used to compare the differential expression of *A. veronii* between the biofilm and planktonic cells. The results show alterations in 277 proteins, with 130 being upregulated and 147 downregulated. Pathway analysis and GO (Gene Ontology) annotations indicated that these proteins are mainly involved in metabolic pathways and the biosynthesis of secondary metabolites and antibiotics. These proteins are the main factors affecting the adaptability of *A. veronii* to its external environment. MRM (multiple reaction 27 monitoring) and qPCR (qPCR) were used to verify the differential proteins of the selected *A. veronii*. This is the first report on the biofilm and planktonic cells of *A. veronii*, thus contributing to studying the infection and pathogenesis of *A. veronii.*

## 1. Introduction

*Aeromonas veronii* is a virulent Gram-negative pathogen associated with infections in freshwater fish species and mammals [1,2]. *Aeromonas* spp. are capable of adhering to biotic or abiotic surfaces that are surrounded by an extracellular matrix produced by the resident microorganisms [3]. To adapt to the natural environment, the biofilm is often adsorbed onto biomaterials or the surface of body lumens (such as plastics, metals, glass, soil, etc.) to secrete alginate polysaccharide and lipid proteins among others [4,5,6,7]. Several factors such as bacterial defense mechanisms, suitable areas for colonization, community cooperation-related benefits, and their extraordinary mode of growth in their habitat, could accelerate biofilm formation [8,9]. Moreover, biofilm plays an important role in bacterial survival mechanisms and antibiotic tolerance [10]. Biofilm can act as a physical barrier against drugs and provide a protective ecological niche for the survival of microorganisms. In recent years, one of the most important reasons for the difficulty in treating clinical infections is the formation of biofilm on wounds [11]. Biofilm formed on planktonic cells is accompanied by changes in physical and chemical properties, such as increased resistance to UV light and genetic exchange efficiency, the ability to degrade macromolecules, and increased yield of secondary metabolites [12]. Moreover, a few studies have indicated biofilm to be drug-tolerant or to facilitate persistent cell survival in the presence of antibiotics, resulting in difficulties in eliminating bacteria [13]. The properties of bacterial biofilm provide several advantages over planktonic growth, especially increased resistance to antibiotics [14]. Not only the formation of biofilm but also the differences between biofilm and planktonic cells have been focused on.

At present, iTRAQ stands out from traditional proteomics technologies because of its unique advantages, such as accurate quantification, high-efficiency sample separation, a high identification rate, and excellent instrument performance [15]. iTRAQ analysis has been further strengthened by powerful bioinformatics tools and statistical analyses [16]. So far, few works have studied the genes and proteins involved in *A. veronii* biofilm formation [17]. In this study, the effects of different media and culture time on the biofilm formation of *A. veronii* were investigated to determine the optimized conditions for biofilm formation. Moreover, the biofilm formation ability was observed by scanning electron microscopy. The proteins that were differentially expressed between the planktonic and biofilm states of *A. veronii* were screened using iTRAQ-based proteomics analysis, which might identify new potential proteins for the development of vaccines and inhibitors of biofilm formation.

## 2. Results

### 2.1. The Optimization of Biofilm Formation Conditions

**Figure 1:**
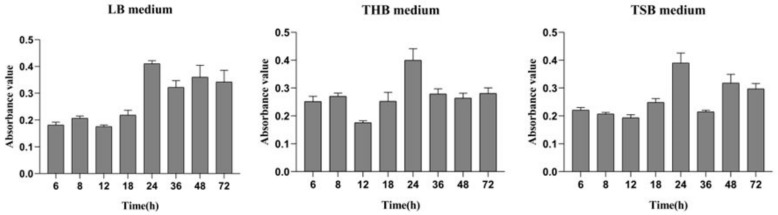
Differential media for *A. veronii* biofilm formation.

**Figure 2:**
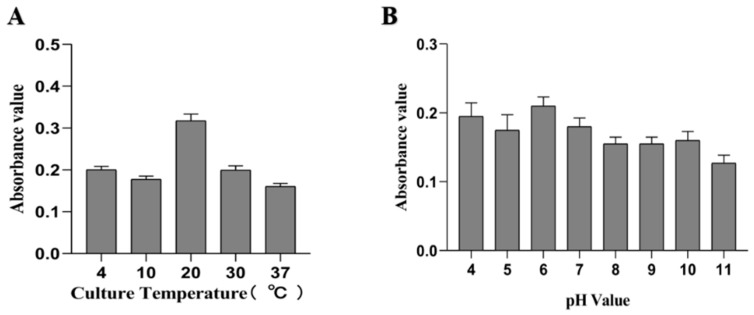
Differential culture temperature (**A**) and pH (**B**) for *A. veronii* biofilm formation.

**Figure 3:**
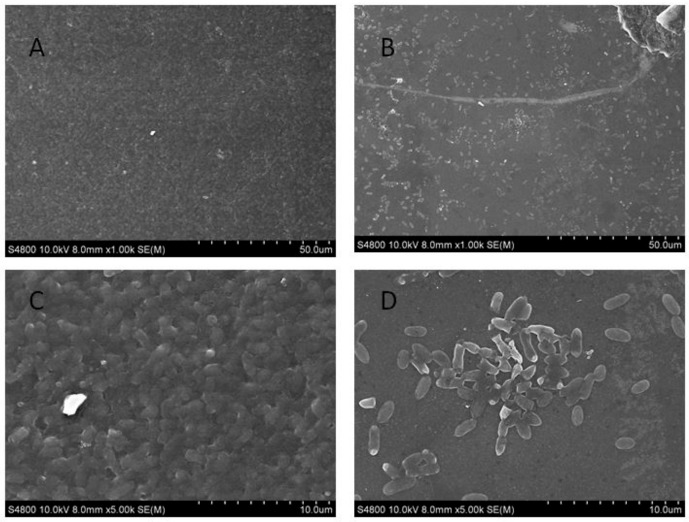
Scanning electron microscopy used to observe *Aeromonas veronii* TH0426 biofilm formation. (**A**) biofilm state bacteria (×1000); (**B**) planktonic state bacteria (×1000); (**C**) biofilm state bacteria (×5000); (**D**) planktonic state bacteria (×5000).

From the results determined by absorbance at 575 nm, it can be seen that the optimal time for biofilm formation was 24 h (Figure 1). With an increase in culture time, biofilm formation was in a fluctuating state, and different culture media had no significant effects on biofilm formation. Biofilm formation at 20 °C was significantly higher than that at other temperatures, as shown in Figure 2A. It can be seen from Figure 2B that the pH corresponding to the highest amount of observed biofilm formation in the assayed conditions was 6 (Figure 2B). Using SEM, it could be observed that *A. veronii* grew uniformly on the surface of the cover slide, forming a dense membrane-like substance. The biofilm of *A. veronii* covered the slide with far more bacteria than the plankton, ultimately covering the entire cover slide (Figure 3).

### 2.2. Planktonic Cells and Biofilm Protein Extraction State

**Figure 4:**
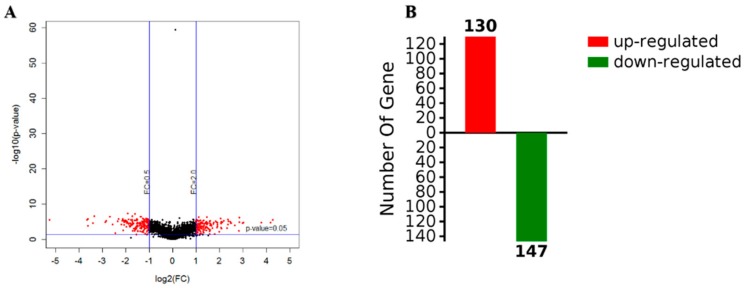
Volcanic maps of gene expression (**A**) and the number of upregulated and downregulated proteins (**B**).

The first planktonic cell organisms to be extracted were in a protein film state, and the application of a BCA protein assay kit was used for quantitative analysis; the abscissa is the concentration of protein (mg/mL), and the ordinate is the absorbance value of the standard curve (Figure 4A). The R^2^ value of the standard curve was 0.9945, indicating its credibility, and the lowest concentration of 4.072 mg/mL in the samples met the necessary requirements.

The extracted protein was separated using a 12% SDS-PAGE gel. Each lane was loaded with a total of 10 g protein corresponding to a particular sample. There were no significant differences between the three replicates of the two groups, and the quantitative consistency was good, indicating that the protein extraction between the samples was consistent, as shown in Figure S1.

### 2.3. Differential Protein Expression Analysis

The screening criteria for the differential proteins were as follows: The fold change was 2 times, and the *p*-value < 0.05. According to a *t*-test, a protein with a *p*-value < 0.05 was considered to be significantly different, and a protein with multiple changes greater than 2 or less than 0.5 was considered to be a differential protein. According to the results, a volcano map was constructed, where red is used to represent differential data points (Figure 4A). The abscissa of the volcanic map was log2 (in fold change); the farther the distance from 0, the greater the difference. The vertical coordinate was log10 (*p*-value); the smaller the *p*-value value, the more significant the difference, while the farther the ordinate value is from 0, the bigger the difference. Each point in the volcano map represents a protein. The red dots in the upper left corner and the upper right corner represent the screened differentially expressed proteins, and the black spots are non-differential proteins. A total of 2248 proteins were obtained with high confidence, with 277 differentially expressed proteins observed for biofilm and planktonic state bacteria, as shown in Table S1, which shows the upregulation of 130 proteins (red), including a protein kinase for TonB biological transport protein as well as 3-nucleoside and two phosphate dehydrogenases, and downregulation of 147proteins (green), including heat shock protein (including flagellin) and PTS sugar transporter (Figure 4B).

### 2.4. GO Annotation

**Figure 5:**
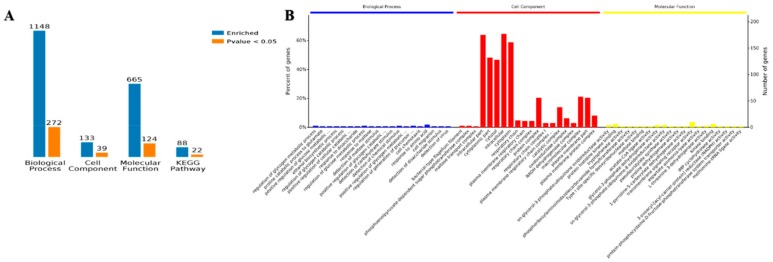
Gene Ontology (GO) annotation of the differentially accumulated proteins of the planktonic and biofilm states. (**A**) The number of enrichment proteins with GO annotations and Kyoto Encyclopedia of Genes and Genomes (KEGG). (**B**) The percentage of proteins with GO annotation.

Functional enrichment analysis of the Gene Ontology (GO) annotations for the biological process was used to show the involved biological processes of 1148 items identified in planktonic cells and biofilm as corresponding genes that were differentially and 272 proteins that were significantly (*p* < 0.05) expressed. Moreover, 133 proteins were identified as being involved in the cell components, 39 of which were more significant. There were 665 entries involved in molecular function, and 124 entries were more significant. There were 88 entries in the Kyoto Encyclopedia of Genes and Genomes (KEGG) pathway analysis, and 22 of them were significant (Figure 5A).

The GO enrichment analysis overview shows that Biological Process (BP), Cell Component (CC), and Molecular Function (MF) were the top twenty entries for three kinds of enrichment analyses. Cell fragments and cytosol were found for the cell components of the proteins, with cytoplasm being the main location. The amino acid biological process showed the greatest number of proteins, and the transmembrane signal receptor activity was involved in the molecular function of most proteins. The enrichment analysis of biological processes, cell components, and molecular components are shown in Figure 5B. The details of the GO enrichment analysis are shown in Figure S2–S4.

### 2.5. Pathway Analysis

The KEGG analysis found that 74 genes were associated with the biosynthesis of secondary metabolite accumulation, accounting for a total percentage of 26%. The NDK genes located within the cell were also involved in this pathway. The 1plA genes were found to be involved in the metabolic pathway; this pathway accounts for a total percentage of 19% (132 genes with enrichment).The value of *p* < 0.01 for signaling pathways indicates the significance of pathways, including naphthalene chloride alkyl and chlorinated olefin degradation, and a significant ranking for the bacterial chemotaxis pathway (Figure S5).

### 2.6. Analysis of Protein–Protein Interactions (PPI)

**Figure 6:**
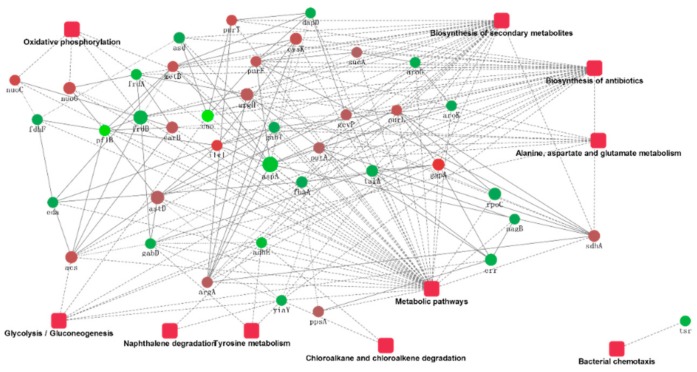
The protein–protein interaction network.

The ten proteins’ significant combinations in the KEGG pathway, as well as the protein interactions, are plotted in Figure 6. The protein interaction networks have been graphed as mutual maps with a dot (gene or protein) and connected dots (showing the interactions; the dashed lines have not been verified by experiments and solid lines have been verified by reports), as well as dots and rectangular fillets (showing biological processes, cellular locations, molecular functions, or signal pathways), connections (associations and participation), and color changes (red and green indicate the upregulated and downregulated expression, respectively.), showing the network and molecular mechanisms for the interactions of the data display. Secondary metabolite biosynthesis, metabolic pathways, antibiotic biosynthesis, alanine, aspartate, and glutamate metabolism interact with a variety of proteins, and the bacterial chemotaxis pathways interact only with downregulated *tsr* genes. The *sdhA* gene involved in the biosynthesis of secondary metabolites and other genes involved in the upregulation of *argA*, *purF*, and *purL* were confirmed to interact with downregulated *nagB* genes.

### 2.7. Verification of Differential Protein

**Figure 7:**
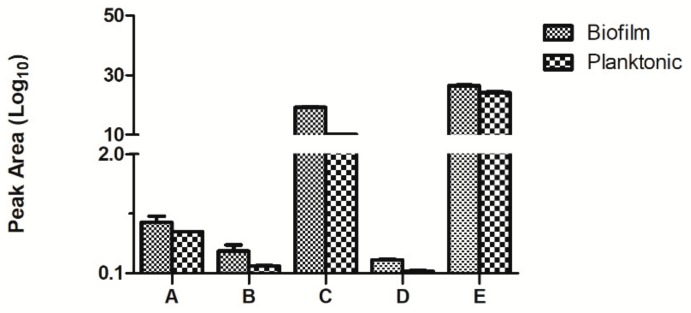
Comparison of the multiple reaction monitoring (MRM) results of the differential proteins in the planktonic and biofilm states of *Aeromonas veronii* TH0426 (A: biopolymer transporter TonB. B: glycerol-3-phosphate dehydrogenase. C: nucleoside diphosphate kinase. D: hypothetical protein AO720_15660. E: Porin).

The accuracy of this test was verified by multiple reaction monitoring (MRM). We randomly selected five of the differently upregulated proteins, namely biotransport of TonB, 3- phosphoric glycerol dehydrogenase, nucleoside diphosphate kinase, the hypothetical protein AO720_15660, and pore protein. The results are shown in Figure 7. The results corresponded with those of iTRAQ, giving support to the iTRAQ results in accurately reflecting the in vivo situation.

### 2.8. Verification of the Fluorescence Quantitative PCR Results

**Figure 8:**
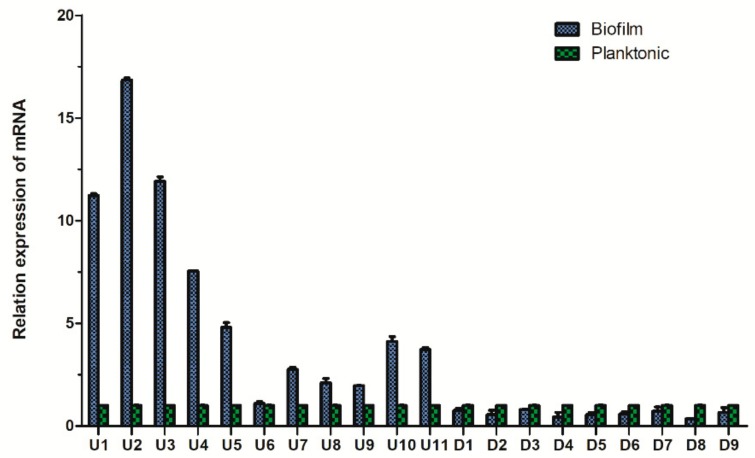
Comparison of the fluorescence quantitative PCR results of the differential proteins of *Aeromonas veronii* TH0426 in planktonic and biofilm states.

We randomly selected 20 differentially expressed proteins (11 upregulated proteins and 9 downregulated proteins). The gene IDs of these proteins are shown in Table S2. The qPCR results showed that the expression trend of the 20 proteins in the two groups was in accordance with the iTRAQ results (Figure 8).

## 3. Discussion

*A. veronii* is capable of infecting a wide range of fish species, resulting in serious damage to the aquatic industry [18]. Many studies have indicated that bacteria that are able to form a biofilm may have stronger pathogenicity and infectivity, such as *Staphylococcus aureus* [19,20]. Thus, the key proteins involved in *A. veronii* biofilm formation have significant importance. A total of 96 microporous culture plates were studied, and the crystal violet staining method was used to quantitatively analyze the bacterial biofilm formation, similar to previous studies [21,22,23]. Moreover, scanning electron microscopy can be used to directly observe the formation of bacterial biofilm, as has previously been carried out for *Staphylococcus aureus* and *Escherichia coli* biofilm [24,25,26]. To investigate the relationship between culture conditions and biofilm formation ability, the temperature, pH, and culture medium were chosen as variables for experimental conditions. The results show that the choice of culture media did not affect biofilm formation, as was the case for a study examining *Aeromonas hydrophila* [27]. Temperature plays a key role in bacterial reproduction and biofilm formation ability [28]. The biofilm formation ability significantly increased at 20 °C, and the biofilm was able to be formed at 4 °C. Moreover, the biofilm formation ability was poorer at 10 °C than at 4 °C. The results differ from those of a previous study on *A. hydrophila* [27]. This difference may have been caused by the different bacterial species assayed and, therefore, proteins associated with biofilm formation. pH was also shown to influence biofilm formation [22], and the results showed that *A. veronii* biofilm formation was significantly different at pH 6. Bacterial biofilm formation is complex and is affected by many factors [22,23]. To explore the different proteins involved in biofilm and planktonic state bacteria, an accurately constructed model of the biofilm state is key.

Proteomics technology has been widely used and has yielded great advances in scientific research [29]. Studies on differential bacterial proteomics have gradually deepened [29,30,31], but studies on the differentially expressed biofilm and planktonic cell proteins of *A. veronii* have not yet been reported. In this study, iTRAQ-based technology was used to analyze the proteins whose abundances differed significantly between the biofilm and planktonic cells of *A. veronii* [32,33]. A total of 277 differentially expressed proteins were identified between the biofilm and planktonic states of *A. veronii*, including 130 upregulated and 147 downregulated proteins. Among the 130 upregulated proteins, there were some significantly different proteins, including the biological transport of TonB protein, the gene encoding *yebC*, and the *lplA* gene encoding the putative protein AO720_15660. The TonB system is essential for the uptake of important nutrients from the environment [34]. This system is anchored in the intima ExbB–ExbD, and the periplasmic protein TonB constitutes the TonB dependent outer membrane receptors (TBDTs) that provide energy for transporting nutrients [34,35]. iTRAQ results showed ExbB was significantly upregulated. It indicated the TonB–ExbB pathway played an important role in the biofilm formation of *A. veronii*. Moreover, the TonB system is generally involved in the transport of iron heme, vitamin B12, carbohydrates, and a variety of transition metal elements, as well as other important substances [35,36,37]. So, it could be inferred that bacteria take in nutrition for biofilm formation and maintaining biofilm state through TonB–ExbB. Further, the *lplA* gene encoding upregulated protein AO720_15660 plays a key role in bacterial adhesion and maintaining a bacterial steady-state [38,39]. Upregulated *lplA* gene may limit bacterial motility through adhering to each other to affect bacterial biofilm formation. Flagellin, an important component of flagellum, was one of 147 downregulated proteins. Moreover, bacterial flagella are the movement organs but are also important virulence factors [40], and flagellar filaments are encoded by the *fla* gene in *A. veronii* TH0426 [1]. While in a Salmonella enteritidis study, the *fliC* gene encoded them, and the study showed the formation ability of *fliC* gene deletion strain was poorer than that of the wild strain [41], that indicated *fliC* gene plays an important role in the formation of biofilm [42]. However, the iTRAQ results showed the expression of the biofilm flagellin of *A. veronii* TH0426 was lower than that in the planktonic state. This may shut off the motility of *A. veronii* TH0426 and make it easier for bacteria to stick and construct biofilm. These different results also may be related to a certain difference in the regulation mechanisms of different strains, but the specific reasons for this difference need to be further explored.

The nine differential proteins were identified to have roles in the metabolic pathway, and five proteins were involved in the biosynthesis of secondary metabolites. Further, three proteins were found to be involved in microbial metabolism in various environments. The glyceraldehyde-3-phosphate encoded by the *gapA* gene is involved in seven pathways, including the metabolic pathway, the biosynthesis of secondary metabolites, microbial metabolism in different environments, antibiotic biosynthesis, glycolysis, carbon metabolism, and amino acid biosynthesis. Glyceraldehyde-3-phosphate is the key enzyme in the glycolysis pathway, which is not only involved in membrane transport but can also promote membrane fusion. When distributed on the cell membrane, it also has an adhesion function but can additionally participate in autophagy, promote cell apoptosis, and recruit transferrin [43]. Adhesion, as the first step of bacterial infection, is of great significance to the invasive ability of bacteria and the effective use of toxins [44]. The results showed that the degree of expression in the biofilm state is higher than that in a planktonic state. The outer membrane proteins are located at the outermost surface of the bacteria; they are an important adhesion factor and are also the most likely to become an immunogen that triggers a host’s immune response. These proteins are thus an important pathogenic factor. In this study, expression in the biofilm state was higher than that in the planktonic state, which indirectly indicates that the virulence of the biofilm state of *A. veronii* may be stronger than that in the planktonic state. Moreover, these results may prove bacterial adhesion was stronger, and it could be inferred that bacterial motility may lower and it may be right that flagellin could be downregulated.

By using the KEGG database, we found that enolase encoded by the enolase gene participated in 12 pathways in different environments (including microbial metabolism, metabolic pathway, biosynthesis of secondary metabolites, antibiotic biosynthesis, glycolysis pathway, methane metabolism, carbon metabolism, RNA degradation, amino acid biosynthesis, terpenoid quinone biosynthesis (such as ubiquinone), terpenoid skeleton biosynthesis, and selenium compound metabolism). Enolase is a key enzyme in the glycolysis pathway and a multifunctional protein that participates in the tissue invasion and transfer process of various pathogenic organisms to the host [45,46]. Enolase is widely involved in the regulation of a variety of pathways; however, whether there is any liaison between these needs further research. Several important proteins, related processes, and metabolic pathways were found to be involved in the OXY fitness for biofilm status [47,48,49,50]. *A. veronii* TH0426, a facultative anaerobic [1], changed related metabolic pathways for adaption and survival. Between the biofilm state and planktonic state of *A. veronii* TH0426, pathways were differential, especially in metabolic pathways, and thus indicated that planktonic state should secrete virulence factors for infection and growth. While the biofilm state just needs energy for maintaining a steady state.

We analyzed the protein interactions of several proteins and found that these proteins were mainly involved in metabolic pathways, biosynthesis of secondary metabolites, and biosynthesis of antibiotics. The biofilm formation protein with the highest expression was involved in the metabolic processes of several important proteins either directly or indirectly, and there was a certain link between these proteins. The GO and KEGG enrichment analysis results were basically the same. Thus, we determined that the metabolic pathways involved in these proteins were likely to affect the biological and major factors of planktonic cell differences. This needs to be verified in a future follow-up test. In addition, although no obvious similar rules were found for the downregulated proteins, these differentially regulated proteins may be related to the biological characteristics of different virulent strains.

## 4. Materials and Methods

### 4.1. *Bacterial Strains and Media Preparation*

The isolation of *A. veronii* TH0426 from farmed yellow catfish (*Pelteobagrus fulvidraco*) was performed as described in our previous study, and colonies were picked directly from the RS agar medium (Solarbio, Beijing, China) and inoculated into a liquid Luria–Bertani (LB) medium (Solarbio, Beijing, China) and cultured at 30 °C with shaking for growth of the planktonic state.

### 4.2. Optimization of Biofilm Formation Conditions

To explore the best conditions for biofilm formation, *A. veronii* TH0426 was cultured in different media (LB, Todd-Hewitt Broth medium ( THB ), and Tryptic Soy Agar medium ( TSB ) ) at different pH values (pH 4–11) for different durations (6, 8, 12, 18, 24, 36, 48, and 72 h). The biofilm formation ability was assayed using a modification of the microplate method, according to Stepanovic [51]. The detailed operation steps were as follows. First, a single colony was chosen from an RS agar plate and inoculated into an LB medium and cultured at 30 °C with shaking at 180 rpm for 12 h. Then, the concentration of the bacterial fluid was diluted to 2 × 10^8^ Colony-Forming Unit ( CFU ) /mL with different media at a dilution of 1:100, and 200 μL was inoculated into 96-well plates for culturing with different media, at different times, and under different pH values without shaking. After incubation, the culture media was discarded, and each well was washed three times with 250 μL phosphate-buffered solution (PBS, Solarbio, Beijing, China). Then, 200 μL 99% methanol (Sigma, Darmstadt, Germany) was fixed in each well, and plates were allowed to dry at room temperature. Plates were stained with 200 μL 2.5% crystal violet stain solution (Solarbio, Beijing, China) for 10 min and washed with ultra-water until the excess staining was rinsed off. After the plates were allowed to dry at room temperature, 160 μL 33% glacial acetic acid (Sigma, Darmstadt, Germany) was added into each well, and the OD was measured at 575 nm using a Thermo Scientific Multiskan FC (Thermo Fisher Scientific, Foster city, CA, USA).

### 4.3. Scanning Electron Microscope

To observe the biofilm formation of *A. veronii* TH0426, coverslips were placed into 6-well plates, and the concentration of the bacterial fluid was diluted to 2 × 10^8^ CFU/mL with the optimal medium and temperature without shaking. The medium alone was a negative control, and the planktonic state bacteria were cultured under the same conditions with shaking at 180 rpm. The coverslips were then washed with PBS and fixed with 2.5% glutaraldehyde. Before SEM observation, the coverslips were treated as follows: The samples were first washed 3 times with 0.1 M PBS and then were stained with 2% osmic acid and washed with 0.1 M PBS. The samples were then dehydrated using successive incubations with 30%, 50%, 70%, and 90% ethanol for 15 min, lastly using 100% ethanol for dehydration twice for 15 min. After serial dehydration, digital images of the bacterial cells of the *A. veronii* TH0426 biofilm were subjected to SEM (EVO-18, Zeiss, Jena, Germany) at magnifications of 1000× and 5000×.

### 4.4. Protein Extraction

For bacterial protein extraction, a lysis solution and protease were added into 0.1 g biofilm and *A. veronii* TH0426 prepared in a planktonic state. Ultrasonication was performed at 4 °C on ice for 3 min (80 W, on 0.8 s and off 0.8 s). The substance was centrifuged at 12,000× *g* for 10 min at 4 °C, and the supernatant was collected. Cooled acetone at was added at −20 °C for 2 h and the mixture centrifuged at 12,000× *g* for 10 min at 4 °C; the pellet was then collected. This step was repeated one more time. The pellet was then dissolved in a lysis solution and the solution was centrifuged at 12,000× *g* for 15 min at room temperature. The supernatant was collected and centrifuged again. The resulting supernatant was the extracted protein solution. The concentrations of the protein extracts were determined by the BCA method and stored at −80 °C for iTRAQ analysis.

### 4.5. iTRAQ Labeling and 2D LC–MS/MS Analysis

SDS-PAGE was used to determine the integrity of the proteins and to assess whether the samples should be used for further experiments. A total of 10 μg of proteins from either biofilm and planktonic state *A. veronii* TH0426 were mixed with a 4× protein SDS-PAGE loading buffer (Takara, Dalian, China) and boiled for 10 min. The samples were subjected to separation using 12% SDS-PAGE and stained with Coomassie brilliant blue R250 (Sigma, Darmstadt, Germany). Protein labeling was performed according to the manufacturer’s protocol and a previous study [52,53]. Based on filter-aided sample preparation (FASP), the protein was digested. The samples were labeled by an iTRAQ reagent dissolved with iso-propyl alcohol (Sigma, Darmstadt, Germany); the labeling reaction was stopped by water, and the samples were freeze-dried with a vacuum concentrator. The labeled peptides were then mixed and graded by an Agilent 1200 HPLC system with an Xbridge Peptide BEH C18 column (5 μm, 130 Å, 4.6 mm × 100 mm, Waters). The chromatographic column was balanced by solvent A (10 mM HCOONH4, 5% CAN, pH 10), and the sample was separated from the manual injector to a chromatographic column. Solvent B was 10 mM HCOONH4, 85% CAN, pH 10. The flow rate was 0.3 mL/min. The liquid phase gradient is shown in Table S3.

The eluted peptides were analyzed by a Q-Exactive mass spectrometer (Thermo Fisher Scientific, Waltham, MA, USA). The peptide samples were dissolved in Nano-RPLC Buffer A (0.1% formic acid, v/v) controlled online using Easy-nLC 1000 (Thermo Scientific). MS data were acquired using a data-dependent top 10 method, dynamically choosing the most abundant precursor ions from the survey scan (350–1800 *m*/*z*) for HCD fragmentation. The automatic gain control (AGC) target was set to 3 × 10^6^, and the maximum injection time was set to 50 ms. Survey scans were acquired at a resolution of 70,000 at *m/z* 200, and the resolution for the HCD spectra was set to 17,500 at *m*/*z* 200. The isolation width was 2 *m*/*z*. The normalized collision energy was 30 eV.

To handle the data, we used the Paragon algorithm in the Protein Pilot Software algorithm v. 5 (ABSCIEX, USA) and the experimental *A. veronii* database from NCBI.

### 4.6. Bioinformatics Analysis

The Gene Ontology (GO) database (www.geneontolgy.org) was used for the GO analysis of differentially expressed proteins. Then, the Kyoto Encyclopedia of Genes and Genomes (KEGG) pathway database (http://www.genome.jp/kegg/) was used to further categorize altered proteins utilizing the same resources, as presented by a bar chart.

### 4.7. Verification Test

In this study, multiple reaction monitoring was used for the verification of differential expression proteins with Qtrap 6500 mass spectrometer (AB Sciex, USA), and the gradients used were as follows: 0~3 min, 5% B, 5 min, up to 10% B, 27 min to 15% B, 60 min to 40% B, 61 min to 80% B, 80% B to 66 min, 67 min returning to 5% B, and then retained for 90 min. The declustering potential was set as the Skyline predictive value, the Q1/Q3 four-stage rod was set to 0.7 FWHM, and the spray voltage was set at 2500 V, according to spray condition; the curtain gas at 25 p.s.i (pause) was set to 5 ms and the dwell time was set to 30 ms. The collected MRM data were analyzed by Skyline analysis, and the MRM peaks were confirmed. The MRM peak area of each peptide in the different samples was determined and compared quantitatively. The total RNA was extracted by a Simple RNA Extraction kit (Bioflux, Shanghai, China), and cDNA was synthesized using a PrimeScript™ RT reagent kit with a gDNA Eraser (Perfect Real Time) (Takara, Dalian, China). The relative gene expression was assayed using an ABI 7500 (Thermo Fisher Scientific, Foster city, CA, USA) with an SYBR^®^ Premix Ex Taq™ II (Tli RNase H Plus) (Takara, Dalian, China). A total of 20 genes corresponding to the differentially expressed proteins (11 upregulated and 9 downregulated genes) were randomly selected for qPCR. A total of 16 sRNAs were used as internal controls for the analysis. The qPCR primers are shown in Table S2.

## 5. Conclusions

In this study, quantitation (iTRAQ)-based quantitative proteomics methods were used to compare the differentially expressed proteins of *A. veronii*. The results showed that the upregulated TonB protein increased the nutrient absorption capacity, and the enolase gene was involved in multiple pathway regulations leading to enhancement of the bacteria’s ability for invasion and metastasis. This may be the main reason for the ability of *A. veronii* to form biofilm and its enhanced transmission ability. Future gene knockout experiments will be used to study virulence and resistance genes to better understand their biological characteristics as these genes may potentially be targeted when developing therapeutic drugs. In summary, our study provides new insights into the study of the pathogenicity of biofilm and the treatment of clinical infection.

## Supplementary Materials

The following are available online at https://www.mdpi.com/1422-0067/21/4/1450/s1.

## Abbreviations

GO	Gene Ontology
MRM	multiple reaction monitoring
BP	Biological Process
CC	Cell Component
MF	Molecular Function
KEGG	Kyoto Encyclopedia of Genes and Genomes
LB	Luria–Bertani medium
THB	Todd-Hewitt Broth medium
TSB	Tryptic Soy Agar medium
CFU	Colony-Forming Unit
