# Supplementary material for: An iTRAQ-Based Comparative Proteomics Analysis of the Biofilm and Planktonic States of Aeromonas veronii TH0426
Source: Int J Mol Sci. 2020 Feb 20;21(4):1450. doi: 10.3390/ijms21041450 (PMC7073075; doi:10.3390/ijms21041450)
Supplement: Supplementary file 1 [file ijms-21-01450-s001.zip › Table S1.pdf]

Table S1. Differential proteins between biofilm and planktonic state of *Aeromonas veronii* (Orange and green labels indicated up-regulated and down-regulated proteins, respectively.)

Number	Name	Fold Change (Log2)	P-vaule
gi|754707909	NAD-glutamate dehydrogenase [Aeromonas veronii]	3.123148819	6.04118E-05
gi|953969160	bifunctional acetaldehyde-CoA/alcohol dehydrogenase [Aeromonas veronii]	0.377589044	4.62132E-05
gi|953670319	2-oxoglutarate dehydrogenase subunit E1 [Aeromonas veronii]	2.007734975	6.15633E-05
gi|538454498	protein disaggregation chaperone [Aeromonas veronii Hm21]	0.456134911	3.30859E-06
gi|953669618	carbamoyl phosphate synthase large subunit [Aeromonas veronii]	2.422035115	3.79618E-05
gi|953595055	phosphoenolpyruvate-protein phosphotransferase [Aeromonas veronii]	0.435274285	3.66609E-06
gi|953968979	NAD-dependent malic enzyme [Aeromonas veronii]	0.48051648	2.37231E-05
gi|754674862	lysine decarboxylase LdcC [Aeromonas veronii]	0.469945485	5.76561E-06
gi|491475544	L-cysteine desulfhydrase [Aeromonas veronii]	0.244652406	4.88735E-06
gi|953667844	aspartate ammonia-lyase [Aeromonas veronii]	0.293147122	0.00012014
gi|953669486	RNA helicase [Aeromonas veronii]	2.01992431	2.15848E-06
gi|760138814	aminopeptidase N [Aeromonas veronii]	2.174464457	3.84726E-05
gi|760140176	2',3'-cyclic-nucleotide 2'-phosphodiesterase [Aeromonas veronii]	0.47856704	1.92515E-06
gi|503489007	succinate dehydrogenase flavoprotein subunit [Aeromonas veronii]	2.44356261	2.32526E-05
gi|953973462	phosphoribosylformylglycinamidine synthase [Aeromonas veronii]	2.698739066	0.000303925
gi|953668758	enolase [Aeromonas veronii]	0.138164061	1.421E-05
gi|953972893	arginine--tRNA ligase [Aeromonas veronii]	0.41836455	1.25641E-05
gi|953667323	GTP-binding protein TypA [Aeromonas veronii]	2.018426575	1.45063E-05
gi|953973001	exoribonuclease II [Aeromonas veronii]	2.013708967	0.023105762
gi|953598407	argininosuccinate synthase [Aeromonas veronii]	3.800259516	1.95944E-05

gi|953970162	sn-glycerol-3-phosphate dehydrogenase subunit A [Aeromonas veronii]	0.265649072	5.11635E-08
gi|953669906	transaldolase [Aeromonas veronii]	0.303165781	6.9095E-06
gi|491501999	glycerol-3-phosphate dehydrogenase [Aeromonas veronii]	6.329909665	0.00012836
gi|491487092	phosphoenolpyruvate synthase [Aeromonas veronii]	2.166997372	5.01474E-07
gi|953667008	2,3-bisphosphoglycerate-independent phosphoglycerate mutase [Aeromonas veronii]	0.483566532	7.95066E-05
gi|754673005	hypothetical protein [Aeromonas veronii]	0.478525379	1.65304E-05
gi|760142876	anaerobic glycerol-3-phosphate dehydrogenase subunit B [Aeromonas veronii]	0.197411964	6.84235E-06
gi|491500417	bifunctional phosphoribosylaminoimidazolecarboxamide formyltransferase/inosine monophosphate cyclohydrolase [Aeromonas veronii]	2.674826128	0.000437934
gi|538455549	thymidine phosphorylase [Aeromonas veronii Hm21]	0.406881595	7.83314E-06
gi|754704670	aspartate aminotransferase family protein [Aeromonas veronii]	0.247391209	4.97348E-06
gi|953603955	uridine phosphorylase [Aeromonas veronii]	0.486976924	0.002599379
gi|491502148	NADH dehydrogenase subunit G [Aeromonas veronii]	3.779968609	5.94411E-05
gi|544814653	argininosuccinate lyase [Aeromonas veronii]	2.37702275	0.001064951
gi|491479558	4-hydroxyphenylpyruvate dioxygenase [Aeromonas veronii]	2.095689511	0.000170403
gi|491497700	phosphogluconate dehydratase [Aeromonas veronii]	0.397226674	0.000219069
gi|754707198	chemotaxis protein CheA [Aeromonas veronii]	0.489174225	2.09745E-06
gi|491480237	alcohol dehydrogenase [Aeromonas veronii]	0.397079581	0.00013223
gi|491477507	hybrid sensor histidine kinase/response regulator [Aeromonas veronii]	2.083552487	5.36724E-05
gi|953970662	NADH-quinone oxidoreductase subunit F [Aeromonas veronii]	4.146930021	1.24577E-06

gi|953669925	2,3,4,5-tetrahydropyridine-2,6-dicarboxylate N-succinyltransferase [Aeromonas veronii]	0.430915998	2.96681E-05
gi|953969910	glycine cleavage system protein T [Aeromonas veronii]	2.046769921	7.31726E-05
gi|953972530	succinylglutamate-semialdehyde dehydrogenase [Aeromonas veronii]	2.18215859	0.001943356
gi|953598040	porin [Aeromonas veronii]	7.294399774	1.24166E-05
gi|491497279	chemotaxis protein [Aeromonas veronii]	0.30754352	2.21589E-05
gi|953971140	DNA polymerase III subunit alpha [Aeromonas veronii]	2.100764706	0.000668348
gi|953662176	dihydroxyacetone kinase [Aeromonas veronii]	0.22902567	1.82845E-06
gi|491485954	methyl-accepting chemotaxis protein [Aeromonas veronii]	0.406933228	0.00020591
gi|953600426	fructose-bisphosphate aldolase [Aeromonas veronii]	0.47367829	0.002886055
gi|491483771	dipeptidase [Aeromonas veronii]	0.341403502	4.36775E-06
gi|953666957	sugar ABC transporter substrate-binding protein [Aeromonas veronii]	2.222777276	0.001139033
gi|754708679	flagellin [Aeromonas veronii]	0.026207037	3.07943E-06
gi|953669624	D-3-phosphoglycerate dehydrogenase [Aeromonas veronii]	0.38375667	1.90763E-05
gi|953660626	D-ribose transporter subunit RbsB [Aeromonas veronii]	0.304906326	3.1357E-07
gi|491489383	catalase/peroxidase HPI [Aeromonas veronii]	0.308656819	2.42689E-05
gi|953973180	L-asparaginase 2 [Aeromonas veronii]	0.273707988	2.06569E-06
gi|754704093	aldehyde dehydrogenase [Aeromonas veronii]	5.521796884	1.21317E-05
gi|491477152	succinylarginine dihydrolase [Aeromonas veronii]	2.762250942	5.25357E-07
gi|953971387	chemotaxis protein CheW [Aeromonas veronii]	0.329212752	7.03893E-08
gi|953972460	alanine dehydrogenase [Aeromonas veronii]	2.174910674	7.19583E-06
gi|953669099	fumarate reductase [Aeromonas veronii]	0.395208832	2.37511E-05
gi|491491320	phosphoribosylamine--glycine ligase [Aeromonas veronii]	2.760458167	3.93184E-05

gi|953971090	succinyl-CoA synthetase subunit alpha [Aeromonas veronii]	0.431481888	2.52726E-06
gi|953670417	ATP-dependent 6-phosphofructokinase [Aeromonas veronii]	0.499418551	5.67673E-05
gi|953970918	RNA helicase [Aeromonas veronii]	2.303801187	4.9881E-05
gi|404628293	phosphoribosylaminoimidazole-succinocarboxamide synthase [Aeromonas veronii AMC34]	2.720707988	1.17523E-05
gi|749007959	NADH-quinone oxidoreductase subunit C/D [Aeromonas veronii]	3.352773185	4.40037E-05
gi|953665400	chemotaxis protein [Aeromonas veronii]	0.397110776	3.50369E-07
gi|749008800	fructose-bisphosphatase [Aeromonas veronii]	2.76683087	6.43611E-05
gi|953600154	glycerol-3-phosphate dehydrogenase [Aeromonas veronii]	0.236244655	6.69958E-06
gi|953667988	cysteine synthase [Aeromonas veronii]	2.812907811	4.90544E-05
gi|491502035	tricarboxylic transport membrane protein [Aeromonas veronii]	5.216387012	7.89727E-06
gi|953670318	succinate dehydrogenase [Aeromonas veronii]	2.531203805	0.000167095
gi|538455966	D-lactate dehydrogenase [Aeromonas veronii Hm21]	2.186836138	4.22785E-05
gi|491490502	alanine racemase [Aeromonas veronii]	2.221621867	9.65283E-05
gi|404625748	2-dehydro-3-deoxyphosphogluconate aldolase/4-hydroxy-2-oxoglutarate aldolase [Aeromonas veronii AMC34]	0.480697674	0.000129854
gi|953600174	succinyl-CoA synthetase subunit alpha [Aeromonas veronii]	2.688320841	3.60549E-05
gi|538459176	isocitrate dehydrogenase [Aeromonas veronii Hm21]	2.256349206	4.63247E-06
gi|544814209	dioxygenase [Aeromonas veronii]	4.258736213	1.02611E-05
gi|953970949	universal stress protein UspE [Aeromonas veronii]	0.35073795	3.43946E-06
gi|491480636	membrane protein [Aeromonas veronii]	0.289906587	0.000219721
gi|491488648	amidophosphoribosyltransferase [Aeromonas veronii]	2.519491708	4.3251E-06
gi|404612805	hypothetical protein HMPREF1167_03671 [Aeromonas veronii AER39]	2.391760855	1.37981E-05
gi|754704069	chemotaxis protein [Aeromonas veronii]	0.240303634	1.39593E-05

gi|754708673	flagellar hook protein FliD [Aeromonas veronii]	7.94508761	2.42606E-05
gi|491490376	glyceraldehyde-3-phosphate dehydrogenase [Aeromonas veronii]	5.168422232	2.4472E-06
gi|749006533	4-aminobutyrate transaminase [Aeromonas veronii]	0.477051397	4.89271E-06
gi|953668670	cytochrome d terminal oxidase subunit 1 [Aeromonas veronii]	0.354228495	3.41714E-06
gi|953595261	2-ketocyclohexanecarboxyl-CoA hydrolase [Aeromonas veronii]	0.487951318	2.8436E-05
gi|491490578	pseudouridine-5'-phosphate glycosidase [Aeromonas veronii]	2.146633103	0.000160208
gi|491494206	cytochrome CBB3 [Aeromonas veronii]	2.081000373	0.006735071
gi|953670233	type VI secretion protein [Aeromonas veronii]	0.490502167	0.000230347
gi|953970710	3-deoxy-7-phosphoheptulonate synthase [Aeromonas veronii]	0.425947771	2.18672E-05
gi|953970943	ribose ABC transporter ATP-binding protein RbsA [Aeromonas veronii]	0.452708638	0.000137083
gi|491494856	aryl-phospho-beta-D-glucosidase [Aeromonas veronii]	0.437747315	8.11327E-06
gi|953668190	amino acid ABC transporter substrate-binding protein [Aeromonas veronii]	3.564495139	8.69389E-06
gi|953669154	amino acid ABC transporter substrate-binding protein [Aeromonas veronii]	0.094905239	2.3147E-05
gi|953973032	pyrophosphatase [Aeromonas veronii]	0.35371256	0.000258072
gi|754704563	fructose-6-phosphate aldolase [Aeromonas veronii]	0.431458194	2.23631E-06
gi|953660713	aspartate-semialdehyde dehydrogenase [Aeromonas veronii]	0.481661363	1.75862E-06
gi|953668611	peroxidase [Aeromonas veronii]	2.06224224	6.3701E-06
gi|491491024	PTS trehalose transporter subunit IIBC [Aeromonas veronii]	0.427376186	2.10427E-05
gi|752530607	chemotaxis protein [Aeromonas veronii]	0.352647331	0.000104798
gi|953666549	ribosome-binding factor A [Aeromonas veronii]	2.069060773	3.3835E-06
gi|491493415	threonine aldolase [Aeromonas veronii]	2.087173337	0.000328855
gi|544813372	phosphoribosylglycinamide formyltransferase 2 [Aeromonas veronii]	3.038237335	5.35018E-06

gi|538456814	biopolymer transporter TonB [Aeromonas veronii Hm21]	19.55083565	3.29589E-06
gi|953668150	universal stress protein [Aeromonas veronii]	0.389302454	4.67872E-05
gi|953666498	chemotaxis protein CheW [Aeromonas veronii]	0.463165107	2.2048E-05
gi|749009524	protein PmbA [Aeromonas veronii]	2.746693163	0.00293086
gi|953667985	PTS glucose transporter subunit IIA [Aeromonas veronii]	0.410766333	2.776E-05
gi|953670669	chemotaxis protein CheR [Aeromonas veronii]	0.496572702	5.79022E-06
gi|760138342	DNA phosphorothioation-dependent restriction protein DptH [Aeromonas veronii]	0.495860465	0.000171014
gi|491479303	methyl-accepting chemotaxis protein [Aeromonas veronii]	0.318441312	0.001083831
gi|491498782	sugar ABC transporter substrate-binding protein [Aeromonas veronii]	0.297333098	6.76583E-05
gi|953666548	tRNA pseudouridine synthase B [Aeromonas veronii]	2.654950893	2.63941E-05
gi|953670688	Fis family transcriptional regulator [Aeromonas veronii]	2.847796978	0.000802051
gi|953669072	arginine N-succinyltransferase [Aeromonas veronii]	2.001280475	5.63827E-06
gi|953598553	spermidine/putrescine ABC transporter substrate-binding protein [Aeromonas veronii]	0.469035322	0.000228949
gi|953660708	cytidine deaminase [Aeromonas veronii]	0.29191521	3.3939E-06
gi|754674520	peptide ABC transporter substrate-binding protein [Aeromonas veronii]	3.784288747	0.000186828
gi|404624157	hypothetical protein HMPREF1168_01553 [Aeromonas veronii AMC34]	0.20316633	1.97547E-06
gi|754676313	alcohol dehydrogenase [Aeromonas veronii]	0.469465096	0.000179231
gi|953604081	phosphoribosylaminoimidazole synthetase [Aeromonas veronii]	5.577384999	2.53695E-05
gi|749008844	isovaleryl-CoA dehydrogenase [Aeromonas veronii]	2.645306352	0.000638364
gi|760138921	histidinol-phosphate transaminase [Aeromonas veronii]	2.018334352	0.000192944

gi|538457522	methyl-accepting chemotaxis protein [Aeromonas veronii Hm21]	0.402297619	0.000909043
gi|953668944	chemotaxis protein CheW [Aeromonas veronii]	0.248047908	1.05813E-05
gi|953970116	hypothetical protein [Aeromonas veronii]	2.580798827	0.002306784
gi|953972122	acyl-CoA synthetase [Aeromonas veronii]	3.031279241	0.000124849
gi|491476000	amino-acid N-acetyltransferase [Aeromonas veronii]	2.22481643	0.00043118
gi|953662708	nucleoside diphosphate kinase [Aeromonas veronii]	13.9568254	1.71938E-05
gi|953668630	hypothetical protein AO720_13390 [Aeromonas veronii]	0.348643471	3.08242E-05
gi|953665869	bacterioferritin [Aeromonas veronii]	0.141603287	5.50386E-06
gi|953669953	nucleotide-binding protein [Aeromonas veronii]	0.385810657	2.59904E-05
gi|760140053	alcohol dehydrogenase [Aeromonas veronii]	4.000940734	0.006870162
gi|953668456	cytidylate kinase [Aeromonas veronii]	0.496379968	0.000931282
gi|953670637	cold-shock protein [Aeromonas veronii]	0.221196946	6.75359E-05
gi|760141219	lipoprotein [Aeromonas veronii]	0.457097498	1.22754E-05
gi|953668445	transcriptional regulator [Aeromonas veronii]	0.15653326	3.50968E-07
gi|953969832	trans-2-enoyl-CoA reductase [Aeromonas veronii]	0.184857424	0.016411758
gi|538454251	copper-sensitivity protein C [Aeromonas veronii Hm21]	3.163119594	0.000156565
gi|544812951	D-hexose-6-phosphate mutarotase [Aeromonas veronii]	0.373698139	0.000178691
gi|953667348	C4-dicarboxylate ABC transporter [Aeromonas veronii]	0.27342611	5.32451E-05
gi|749008052	chemotaxis protein [Aeromonas veronii]	0.307163666	0.004385691
gi|953669046	autonomous glycyl radical cofactor GrcA [Aeromonas veronii]	0.170597684	6.86568E-05
gi|953970506	hypothetical protein [Aeromonas veronii]	2.290048136	4.32099E-06
gi|491502762	PTS galactitol transporter subunit IIA [Aeromonas veronii]	0.261017049	2.73791E-05
gi|953603714	orotate phosphoribosyltransferase [Aeromonas veronii]	4.296386719	0.000920377

gi|953669642	hypothetical protein AO720_11930 [Aeromonas veronii]	0.433893532	0.001553583
gi|754674094	MBL fold metallo-hydrolase [Aeromonas veronii]	0.284697509	0.000314012
gi|760139886	molybdate ABC transporter substrate-binding protein [Aeromonas veronii]	0.28336158	2.53881E-05
gi|953662266	oxidoreductase [Aeromonas veronii]	0.424953161	0.000198052
gi|953669675	hypothetical protein AO720_12110 [Aeromonas veronii]	0.266959502	2.06556E-05
gi|953668181	heat-shock protein [Aeromonas veronii]	0.098639642	2.58355E-07
gi|953668027	hypothetical protein AO736_12360 [Aeromonas veronii]	0.347616984	3.88915E-05
gi|328802928	Nitrite reductase [NAD(P)H], large subunit [Aeromonas veronii B565]	0.335736664	0.001972452
gi|953667840	molecular chaperone GroES [Aeromonas veronii]	0.422557718	7.46534E-06
gi|953668628	DNA-binding protein [Aeromonas veronii]	2.381788434	0.000215244
gi|754705400	thiol reductant ABC exporter subunit CydC [Aeromonas veronii]	2.90740988	0.001333291
gi|754671830	dihydroxyacetone kinase [Aeromonas veronii]	0.419847158	0.004654073
gi|503489416	biopolymer transporter ExbB [Aeromonas veronii]	7.124297414	0.004112363
gi|752531175	hydrogenase accessory protein HypB [Aeromonas veronii]	0.455790665	1.29802E-06
gi|953661090	flagellar motor switch protein FliN [Aeromonas veronii]	0.491214388	1.1968E-05
gi|953670677	flagellar biosynthesis protein FlgI [Aeromonas veronii]	2.831806828	2.35094E-05
gi|749006390	hydrogenase expression/formation protein HypE [Aeromonas veronii]	0.343019392	0.001031766
gi|754705249	FMN-dependent NADH-azoreductase [Aeromonas veronii]	4.401415459	1.38483E-05
gi|491492939	flagellar hook-associated protein 3 [Aeromonas veronii]	3.061825483	0.002255519
gi|404627402	dihydroxyacetone kinase, L subunit [Aeromonas veronii AMC34]	0.324221346	3.94309E-06
gi|404619277	hypothetical protein HMPREF1169_00302 [Aeromonas veronii AER397]	0.390818642	3.51586E-07
gi|953666631	cold-shock protein [Aeromonas veronii]	2.059600946	0.00168918

gi|404628179	hypothetical protein HMPREF1170_00440 [Aeromonas veronii AMC35]	0.081722174	2.0144E-06
gi|754704245	sulfurtransferase [Aeromonas veronii]	0.307068748	4.36554E-05
gi|953668623	hypothetical protein AO720_13350 [Aeromonas veronii]	4.543646848	5.36421E-06
gi|953669082	shikimate kinase [Aeromonas veronii]	0.474642958	0.000232943
gi|953970502	RNA chaperone Hfq [Aeromonas veronii]	0.438720295	7.11838E-05
gi|953598692	hypothetical protein AO739_19845 [Aeromonas veronii]	0.489481797	0.00087886
gi|953667591	nitrate reductase [Aeromonas veronii]	0.494292353	0.000561746
gi|953667987	PTS sugar transporter [Aeromonas veronii]	0.08035176	3.03539E-06
gi|953969145	coniferyl-aldehyde dehydrogenase [Aeromonas veronii]	0.406678181	0.007368214
gi|953668489	NADH dehydrogenase [Aeromonas veronii]	3.404801542	2.15774E-05
gi|544815141	chromosome partitioning protein ParB [Aeromonas veronii]	0.305738457	8.49814E-05
gi|953668491	NADH dehydrogenase [Aeromonas veronii]	5.655494126	3.00866E-05
gi|760141875	cytochrome c [Aeromonas veronii]	0.423590628	0.010471744
gi|754673945	formate dehydrogenase [Aeromonas veronii]	0.431484013	0.000605251
gi|754709376	short-chain dehydrogenase [Aeromonas veronii]	7.283154671	8.03496E-07
gi|491498255	acetoacetyl-CoA synthetase [Aeromonas veronii]	2.342175697	0.000217116
gi|953667608	hypothetical protein AO720_15660 [Aeromonas veronii]	18.11596505	2.12493E-05
gi|953667869	hypothetical protein AO720_15100 [Aeromonas veronii]	2.506289905	0.014721528
gi|953665978	ubiquinone biosynthesis methyltransferase UbiE [Aeromonas veronii]	2.006508111	9.56893E-06
gi|953669998	heme utilization protein HutZ [Aeromonas veronii]	2.28097741	2.14733E-05
gi|953971235	nitric oxide dioxygenase [Aeromonas veronii]	0.427397907	0.000297404
gi|538458486	hypothetical protein M001_06835 [Aeromonas veronii Hm21]	3.021721641	1.1639E-05

gi|491502522	hydrogenase 3 large subunit [Aeromonas veronii]	0.441985014	0.00023113
gi|953667898	anti-sigma factor [Aeromonas veronii]	0.319513956	3.31541E-05
gi|754707813	1-phosphofructokinase [Aeromonas veronii]	0.327734384	0.003884712
gi|538455491	hypothetical protein M001_15820 [Aeromonas veronii Hm21]	2.201532194	0.000388266
gi|953668561	hypothetical protein AO720_13005 [Aeromonas veronii]	0.353994545	1.77565E-05
gi|953669571	hypothetical protein AO720_11550 [Aeromonas veronii]	0.338992131	0.000701796
gi|491495583	3,4-dihydroxy-2-butanone-4-phosphate synthase [Aeromonas veronii]	8.183592559	2.11787E-05
gi|491478165	mannitol-1-phosphate 5-dehydrogenase [Aeromonas veronii]	0.382564625	0.006005225
gi|953661049	cell division protein BolA [Aeromonas veronii]	2.126426443	0.00188086
gi|754705208	histidinol dehydrogenase [Aeromonas veronii]	2.168618989	0.003266666
gi|953667185	amidase [Aeromonas veronii]	0.416547638	5.61964E-05
gi|491500461	molybdopterin-binding oxidoreductase [Aeromonas veronii]	0.348837681	1.53543E-06
gi|760144496	glyoxalase [Aeromonas veronii]	0.430863381	0.000464421
gi|953670612	glucosamine-6-phosphate deaminase [Aeromonas veronii]	0.479222786	0.034546641
gi|953973307	RNase E specificity factor CsrD [Aeromonas veronii]	2.234430149	0.003325814
gi|749008612	hypothetical protein [Aeromonas veronii]	2.648407281	2.99748E-05
gi|538457028	ribosome modulation factor [Aeromonas veronii Hm21]	0.229040243	2.11435E-06
gi|491502562	formate dehydrogenase subunit alpha [Aeromonas veronii]	0.488313836	2.35895E-05
gi|538455770	hypothetical protein M001_14890 [Aeromonas veronii Hm21]	0.367673677	2.43222E-05
gi|754705495	acetolactate synthase [Aeromonas veronii]	4.214960449	0.000458109
gi|754672761	glutathione S-transferase [Aeromonas veronii]	3.692688588	1.59862E-05
gi|953972380	hypothetical protein [Aeromonas veronii]	0.233743409	0.003554944

gi|953668939	cytochrome C biogenesis protein CcmE [Aeromonas veronii]	2.152228807	0.000408262
gi|953667333	phosphoribosylaminoimidazole carboxylase [Aeromonas veronii]	2.346652699	0.000202783
gi|491475896	methionine gamma-lyase [Aeromonas veronii]	2.773469574	0.000396312
gi|754676049	restriction endonuclease [Aeromonas veronii]	2.045590096	0.017195344
gi|953971913	hypothetical protein [Aeromonas veronii]	0.40456796	0.000341597
gi|953970335	hypothetical protein [Aeromonas veronii]	2.785631652	1.67979E-05
gi|491496442	sulfite reductase subunit beta [Aeromonas veronii]	3.258460141	0.000769392
gi|754706720	cysteine desulfhydrase [Aeromonas veronii]	0.22512497	5.40645E-06
gi|749009138	amino acid ABC transporter ATP-binding protein [Aeromonas veronii]	2.562052506	4.52458E-05
gi|754672972	isochorismate synthase [Aeromonas veronii]	0.480010513	0.000487099
gi|953668671	cytochrome d ubiquinol oxidase subunit 2 [Aeromonas veronii]	0.333427516	0.000530382
gi|953661081	cytochrome B [Aeromonas veronii]	2.130137628	0.002138058
gi|754708686	flagellin [Aeromonas veronii]	0.330937155	0.001429159
gi|953970126	hypothetical protein [Aeromonas veronii]	0.35966316	0.005156011
gi|491482992	hypothetical protein [Aeromonas veronii]	0.470844194	0.006725594
gi|953668415	transcriptional regulator [Aeromonas veronii]	2.549873592	0.000154052
gi|953663841	spermidine/putrescine ABC transporter substrate-binding protein [Aeromonas veronii]	0.344730461	0.048299129
gi|953662173	hypothetical protein AO720_05750 [Aeromonas veronii]	2.989148864	0.036103796
gi|953970477	hypothetical protein [Aeromonas veronii]	2.311835761	0.017259293
gi|953971552	haloacid dehalogenase [Aeromonas veronii]	0.346282287	0.000243742
gi|754706706	electron transporter HydN [Aeromonas veronii]	0.430900036	7.97094E-05

gi|953968927	C4-dicarboxylate transporter [Aeromonas veronii]	5.018512111	0.000641312
gi|749007928	glycine dehydrogenase (aminomethyl-transferring) [Aeromonas veronii]	2.291077962	0.000200957
gi|953971901	N-acetylglucosamine-binding protein A [Aeromonas veronii]	3.727881821	0.000550918
gi|953972378	short-chain dehydrogenase [Aeromonas veronii]	2.49400779	0.002544835
gi|760142667	NADH dehydrogenase [Aeromonas veronii]	2.994711052	0.006203459
gi|953971822	molybdopterin-binding oxidoreductase [Aeromonas veronii]	0.289775598	3.34943E-05
gi|754708668	hypothetical protein [Aeromonas veronii]	3.062310911	3.26275E-05
gi|953972693	succinate dehydrogenase, hydrophobic membrane anchor protein [Aeromonas veronii]	2.448470588	0.000304691
gi|953667017	hypothetical protein AO736_15420 [Aeromonas veronii]	0.257474177	0.000253518
gi|953971762	hypothetical protein [Aeromonas veronii]	0.259693517	8.34813E-06
gi|491494083	PTS fructose transporter subunit IIBC [Aeromonas veronii]	0.392656288	0.000524521
gi|953973000	transcriptional regulator ArgP [Aeromonas veronii]	2.122572635	0.013796141
gi|953595596	serine/threonine protein kinase [Aeromonas veronii]	2.128047721	4.31039E-06
gi|953970245	bifunctional proline dehydrogenase/L-glutamate gamma-semialdehyde dehydrogenase [Aeromonas veronii]	2.072662931	0.001481267
gi|953668495	NADH dehydrogenase [Aeromonas veronii]	5.141812865	7.21529E-05
gi|754674529	hypothetical protein [Aeromonas veronii]	4.19886876	1.57207E-05
gi|544814756	multidrug ABC transporter ATP-binding protein [Aeromonas veronii]	2.028675441	0.007031193
gi|953970875	formate hydrogenlyase complex iron-sulfur subunit [Aeromonas veronii]	0.447898987	0.022872349
gi|953660638	50S ribosomal protein L35 [Aeromonas veronii]	2.271978376	0.026577105
gi|953667910	nucleoside transporter NupC [Aeromonas veronii]	0.456878059	0.00063438

gi|760139728	PTS fructose transporter subunit IIC [Aeromonas veronii]	0.383564133	0.000809685
gi|953970090	imidazole glycerol phosphate synthase subunit HisH [Aeromonas veronii]	2.287961576	0.006377835
gi|328806753	hypothetical protein B565_3917 [Aeromonas veronii B565]	2.338376398	0.024424901
gi|953665872	hypothetical protein AO720_18285 [Aeromonas veronii]	4.302193132	1.87881E-05
gi|544815169	hypothetical protein [Aeromonas veronii]	2.507837312	0.000693629
gi|491487701	NAD-glutamate dehydrogenase [Aeromonas veronii]	2.647696477	1.97314E-05
gi|754676361	bifunctional acetaldehyde-CoA/alcohol dehydrogenase [Aeromonas veronii]	0.304074414	3.53475E-06
gi|491483486	aspartate ammonia-lyase [Aeromonas veronii]	0.281499771	0.000175682
gi|491496038	formate dehydrogenase [Aeromonas veronii]	0.470819949	0.000291622
gi|760144547	glycerol-3-phosphate dehydrogenase [Aeromonas veronii]	4.213443715	3.49954E-05
gi|491483502	bifunctional phosphoribosylaminoimidazolecarboxamide formyltransferase/inosine monophosphate cyclohydrolase [Aeromonas veronii]	2.349013738	0.000873806
gi|760137978	4-hydroxyphenylpyruvate dioxygenase [Aeromonas veronii]	2.482215169	0.006663046
gi|754707525	chemotaxis protein [Aeromonas veronii]	0.360109635	0.004121742
gi|491486594	alcohol dehydrogenase [Aeromonas veronii]	0.416920955	1.49152E-06
gi|491482729	phosphopentomutase [Aeromonas veronii]	0.442353048	0.000835395
gi|491491656	methyl-accepting chemotaxis protein [Aeromonas veronii]	0.240177259	8.28104E-05
gi|491504924	pseudouridine-5'-phosphate glycosidase [Aeromonas veronii]	2.484790304	5.83358E-06
gi|754672163	4a-hydroxytetrahydrobiopterin dehydratase [Aeromonas veronii]	3.506145351	0.032610962
gi|491496439	sulfite reductase [NADPH] flavoprotein, alpha-component [Aeromonas veronii]	2.338540359	0.000456462

gi|491499880	aspartate--ammonia ligase [Aeromonas veronii]	2.022631065	0.010620783
gi|953969153	D-serine ammonia-lyase [Aeromonas veronii]	2.28097078	2.68845E-05
