# Supplementary material for: An iTRAQ-Based Comparative Proteomics Analysis of the Biofilm and Planktonic States of Aeromonas veronii TH0426
Source: Int J Mol Sci. 2020 Feb 20;21(4):1450. doi: 10.3390/ijms21041450 (PMC7073075; doi:10.3390/ijms21041450)
Supplement: Supplementary file 1 [file ijms-21-01450-s001.zip › Table S2.pdf]

Table S2. qPCR primers for differential proteins verification

Number	Gene ID	Name	Primer sequence (5'-3')	Tm(°C)
U1	gi|538456814	TonB-F	GCATCCAAAGTTTCCCAGC	57.2
		TonB-R	GCATCCAAAGTTTCCCAGC	57.8
U2	gi|953667608	lplA-F	GAAGGTCATCTTGGGATACTGC	58
		lplA-R	AGGCGAGCTTTGAAGTGGA	58.1
U3	gi|754705495	ilvI–F	TCGGCATAGCGTTTGAAGTC	58.8
		ilvI–R	TGCGGCTCAAGAGCAATATC	58.9
U4	gi|953668495	NADH-F	ATCGTGTTGACCCGTGACC	58.2
		NADH-R	TACCAGCGACCATCTTCCC	58
U5	gi|754704093	F	ACAAGATCCTGGGCTACATGG	59.0
		R	TCCTCCTGGAACACCCTCAT	58.8
U6	gi|754708673	FliD-F	CTCATCGCAGAACCAGTAGCA	58.8
		FliD-R	TGATGTCAAGGCAGGGGAT	58
U7	gi|953668491	F	AACACGAGAAACACCACTACGA	57.4
		R	CGGATAATGTGACGCCCTAC	58.0
U8	gi|754709376	F	GTTCGGACAGTTTGGGTGC	58.3
		R	GCTGCCATCGAGGATTTGA	59.1
U9	gi|491495583	F	GGTAAAGGCGGTCTGGTAGTG	59
		R	GATGAAGATCGGGAGAACGAG	58.7
U10	gi|544814209	F	CTCGTCGGTGAACTTCTTGG	58
		R	GAGCCGCTTCGTGATCTGTA	58.7
U11	gi|953970662	F	TACAGAGCAGGTAACGGATGTTC	58.9
		R	TTGTCAGCACCGTCAAGGAT	58.5
D1	gi|953670669	CheR-F	ACGCCTTCCTTACACTGATTG	57.1
		CheR-R	ACCCTCTGGTTTCGTGATACTT	57.3
D2	gi|95366071	F	AGGGCTACTGGATTGACGC	57.4
		R	AGGTCTTGATGCCATCGTTC	57.2
D3	gi|953661090	FliN-F	ACCAGTAGCAGCAAACAGCG	59
		FliN-R	CCACTTCCAGCGTCACCTT	58.0
D4	gi|749006533	F	CGAGATCCAGAGCGGTTTT	57.6
		R	CTGGTTGAGTTTCTCCTCGTCT	57.8
D5	gi|760140176	F	TTGAACTGGACGGCACTCTT	57.9
		R	TCTATGTCTACCCCAACACCCT	57.9
D6	gi|538454498	F	GCTTTCATCTCGTCGTAATCCTT	59.8
		R	ATTTCCGCAACTCGGTGGT	60.1
D7	gi|953666498	CheW-F	ATCACCCTCAACACCTCCC	57.9
		CheW-	AGCAAATCGGGCTGGAAT	57.7
D8	gi|953971090	F	CCGGCATATCCTTCCACTT	57.3
		R	ATAACTTCGCCACCAACACC	57.4
D9	gi|754674862	LdcC-F	CGGGCTGAAGTTGGTGTAAG	58.2
		LdcC-R	ACGGCATTCTGGGTGGTAT	57.3
16s	-	338F	ACTCCTACGGGAGGCAGCAG	55.7

	518R	ATTACCGCGGCTGCTGG	55.2
