# Supplementary material for: An iTRAQ-Based Comparative Proteomics Analysis of the Biofilm and Planktonic States of Aeromonas veronii TH0426
Source: Int J Mol Sci. 2020 Feb 20;21(4):1450. doi: 10.3390/ijms21041450 (PMC7073075; doi:10.3390/ijms21041450)
Supplement: Supplementary file 1 [file ijms-21-01450-s001.zip › Table S3.pdf]

Table.S3 Peptide chromatographic separation of mobile phase scale

Time(min)	0	3	3.01	40	50	50.01	60	60.01	65
B%	2	2	6	25	38	90	90	2	2
