# Supplementary figures and images for: An iTRAQ-Based Comparative Proteomics Analysis of the Biofilm and Planktonic States of Aeromonas veronii TH0426
Source: Int J Mol Sci. 2020 Feb 20;21(4):1450. doi: 10.3390/ijms21041450 (PMC7073075; doi:10.3390/ijms21041450)

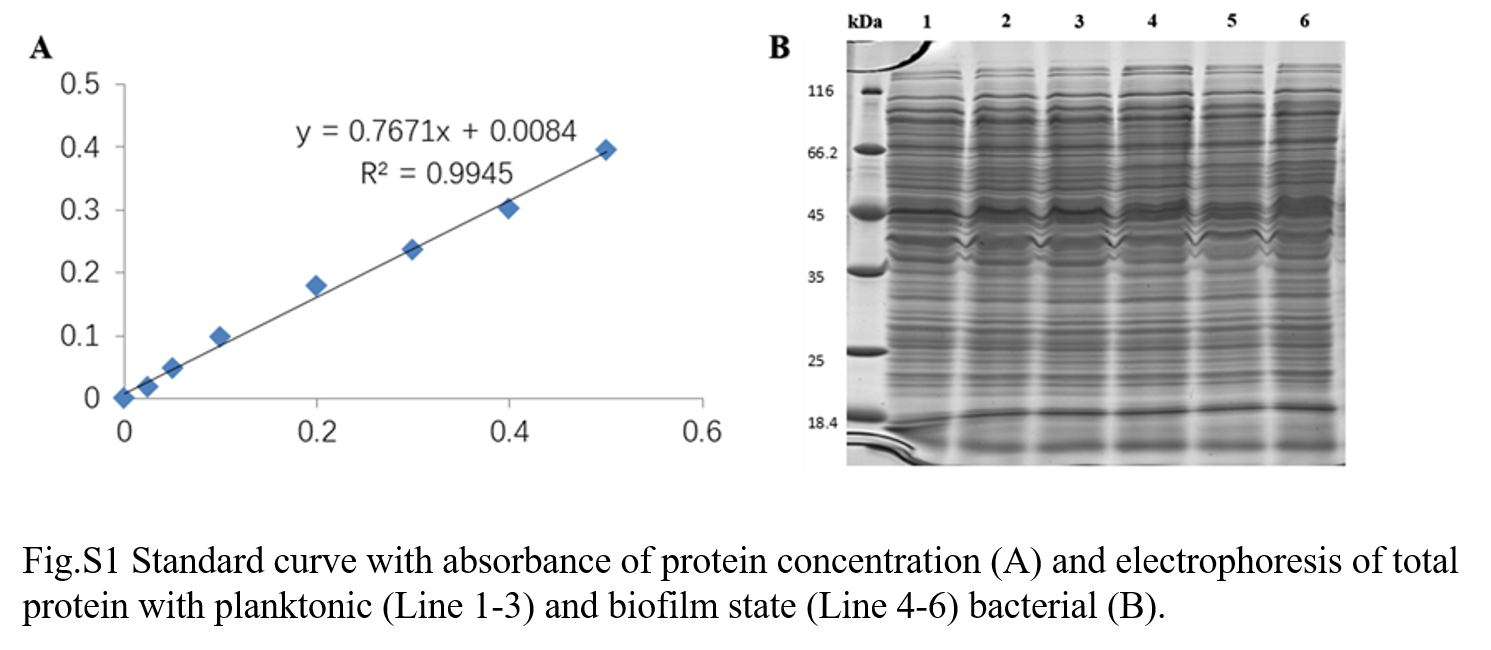

Supplement: Supplementary file 1 [file ijms-21-01450-s001.zip › FigS1.tif]

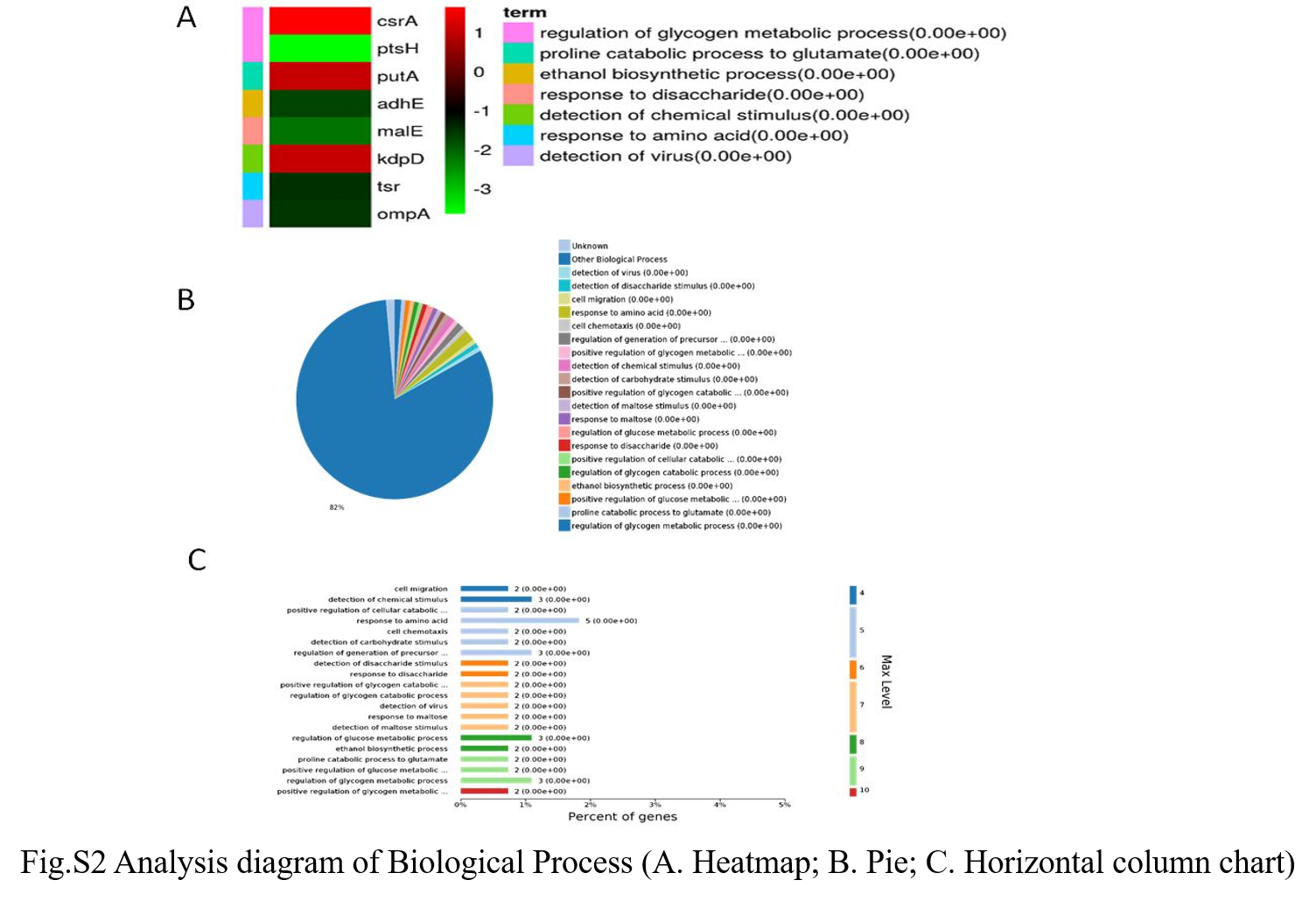

Supplement: Supplementary file 1 [file ijms-21-01450-s001.zip › FigS2.tif]

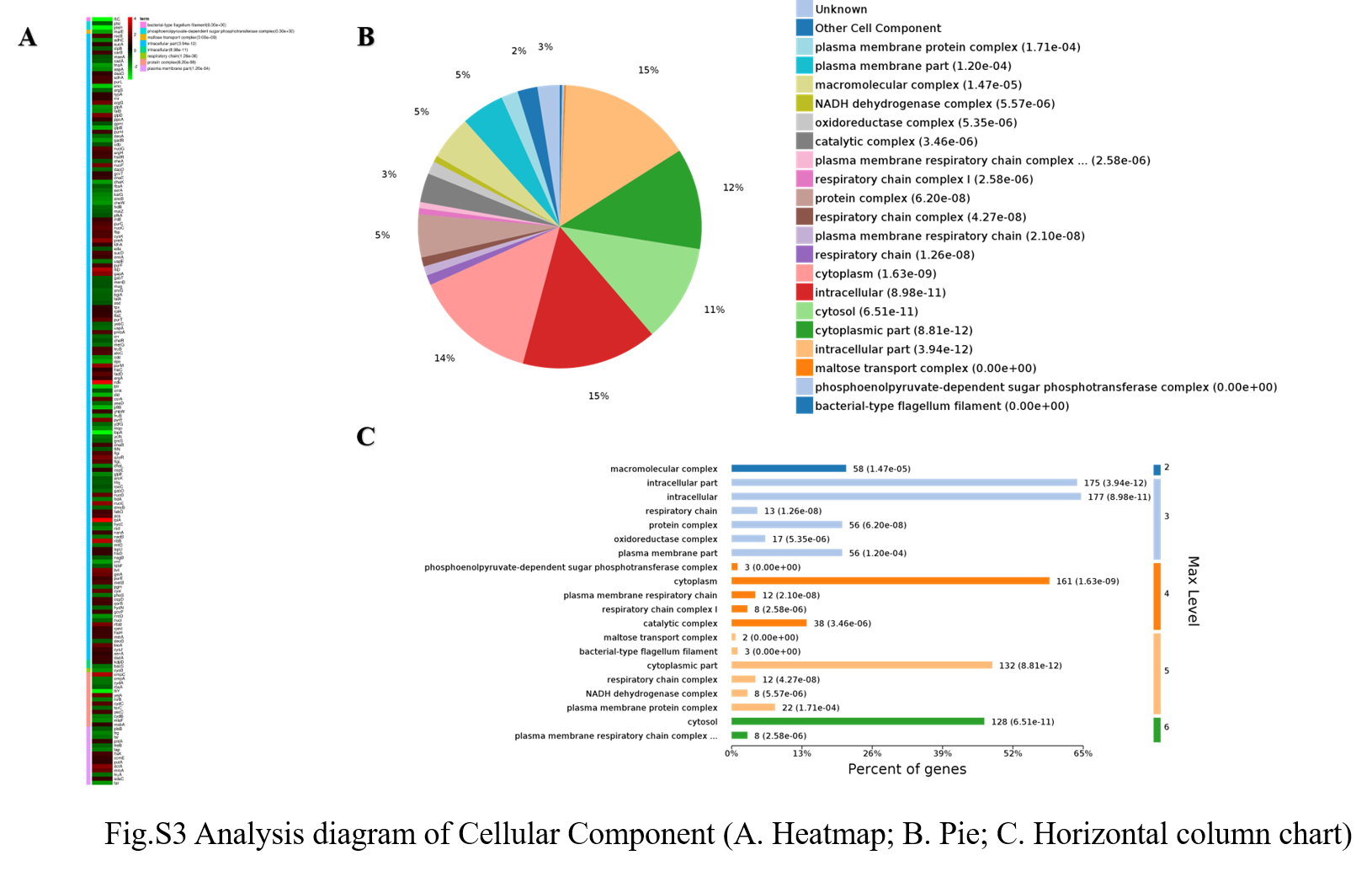

Supplement: Supplementary file 1 [file ijms-21-01450-s001.zip › FigS3.tif]

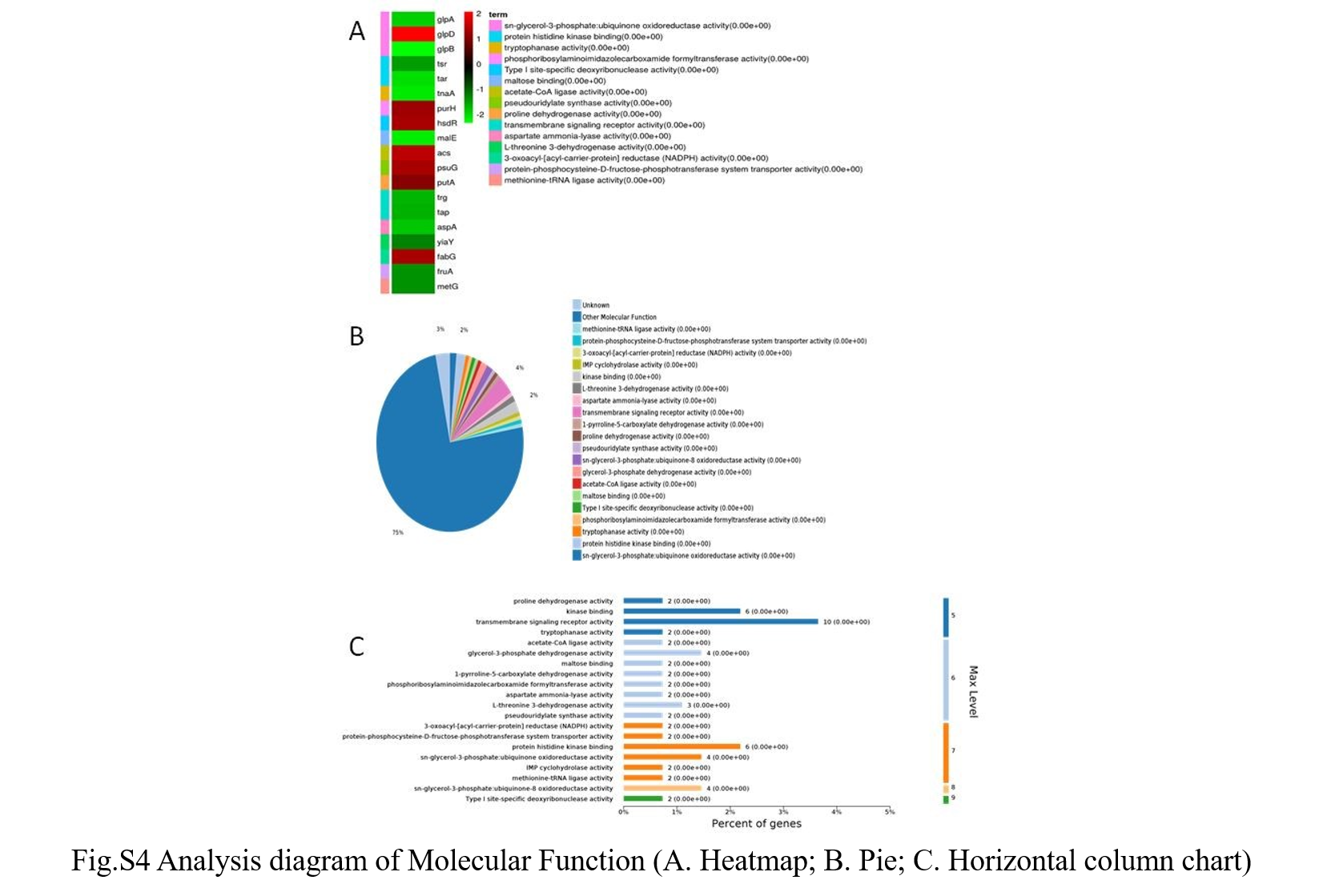

Supplement: Supplementary file 1 [file ijms-21-01450-s001.zip › FigS4.tif]

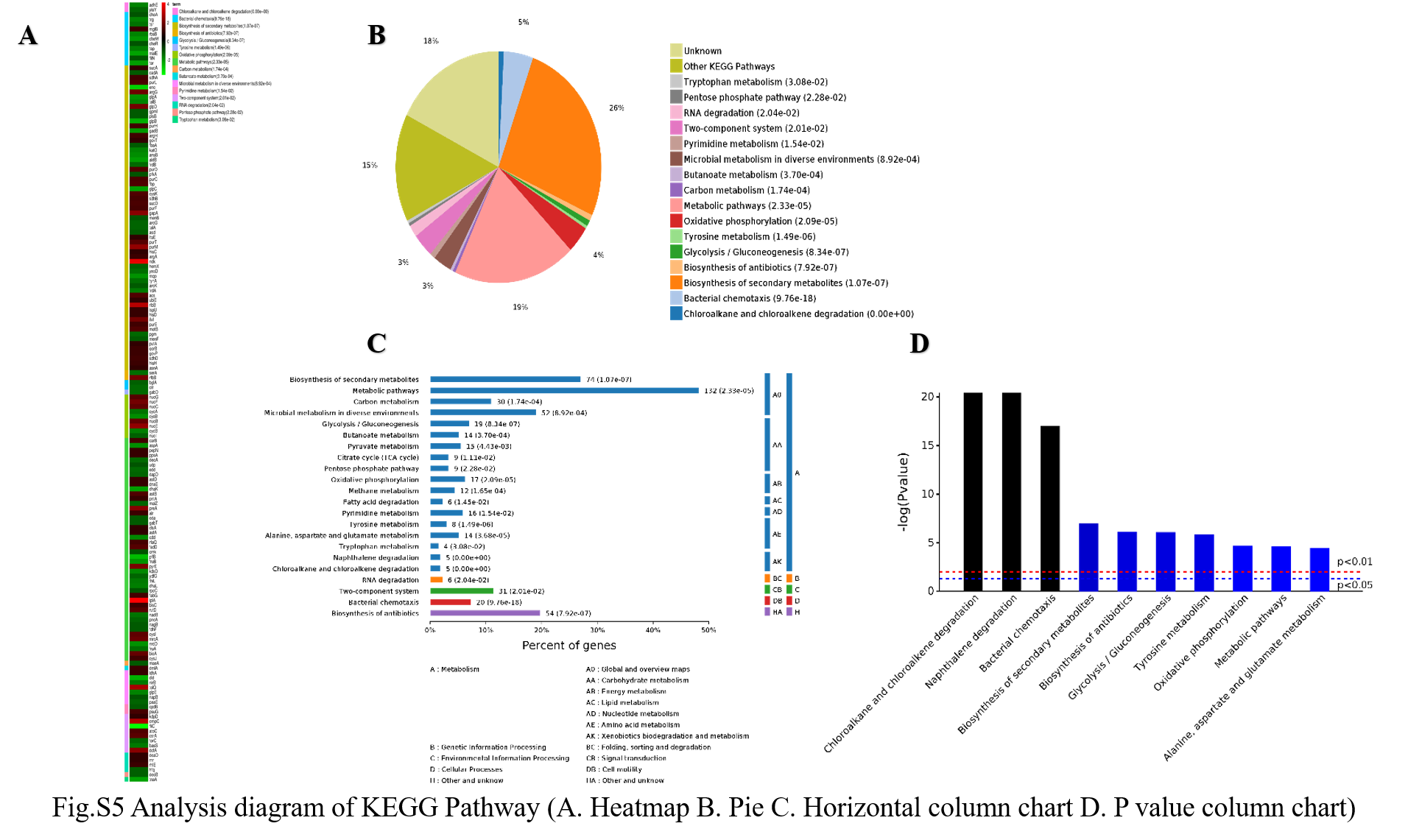

Supplement: Supplementary file 1 [file ijms-21-01450-s001.zip › FigS5.tif]
